# Innovations in Cattle Farming: Application of Innovative Technologies and Sensors in the Diagnosis of Diseases

**DOI:** 10.3390/ani13050780

**Published:** 2023-02-21

**Authors:** Karina Džermeikaitė, Dovilė Bačėninaitė, Ramūnas Antanaitis

**Affiliations:** Large Animal Clinic, Veterinary Academy, Lithuanian University of Health Sciences, LT-47181 Kaunas, Lithuania

**Keywords:** biosensors, precision dairy farming, sensors technology, dairy cattle, early diagnosis

## Abstract

**Simple Summary:**

As the importance of farming continues to grow, innovative technology and sensors play an increasingly important role. Automation and robots in agriculture have the potential to play a significant role in helping society fulfill its future demands for food supply. Wearable sensors connected to or within cows can monitor eating, rumination, pH, body temperature, laying behavior, animal activity, animal position or placement, and more. Research concentrates on biosensing technologies that have the potential to improve livestock early disease detection, management, and operations. The goals of this study were to investigate the currently available knowledge regarding agricultural innovations and their application tactics, and we intended to give a critical perspective and advance the understanding of what is known and unknown about innovations and dairy cattle.

**Abstract:**

Precision livestock farming has a crucial function as farming grows in significance. It will help farmers make better decisions, alter their roles and perspectives as farmers and managers, and allow for the tracking and monitoring of product quality and animal welfare as mandated by the government and industry. Farmers can improve productivity, sustainability, and animal care by gaining a deeper understanding of their farm systems as a result of the increased use of data generated by smart farming equipment. Automation and robots in agriculture have the potential to play a significant role in helping society fulfill its future demands for food supply. These technologies have already enabled significant cost reductions in production, as well as reductions in the amount of intensive manual labor, improvements in product quality, and enhancements in environmental management. Wearable sensors can monitor eating, rumination, rumen pH, rumen temperature, body temperature, laying behavior, animal activity, and animal position or placement. Detachable or imprinted biosensors that are adaptable and enable remote data transfer might be highly important in this quickly growing industry. There are already multiple gadgets to evaluate illnesses such as ketosis or mastitis in cattle. The objective evaluation of sensor methods and systems employed on the farm is one of the difficulties presented by the implementation of modern technologies on dairy farms. The availability of sensors and high-precision technology for real-time monitoring of cattle raises the question of how to objectively evaluate the contribution of these technologies to the long-term viability of farms (productivity, health monitoring, welfare evaluation, and environmental effects). This review focuses on biosensing technologies that have the potential to change early illness diagnosis, management, and operations for livestock.

## 1. Introduction

The future of animal farming will be guided by the principles of precision, sustainability, and intelligence. Accurate cattle production can only be attained with the rapid spread of intelligent technology for early warning of illnesses, feeding precision, and remote diagnosis [1]. In recent years, the dairy sector has developed and deployed a number of different technologies in order to automatically monitor a variety of behavioral and physiological indicators [2,3]. Collecting large amounts of data is made possible by the use of sensors and technology, and these data must be analyzed with sophisticated statistical methods before any conclusions can be drawn about the animals’ behavior, health, or welfare. Innovations and information technologies (ITs) are essential for achieving sustainable operations because they enable early and rapid disease detection, help measure environmental emissions, and optimize production [4].

Traditional diagnostic procedures are notoriously labor-intensive, time-consuming, and technical, necessitating the expertise of skilled specialists using specialized equipment. To overcome these obstacles and create quick-response, low-cost, and highly reliable biosensor devices, new technologies are being employed [5]. The use of automated remote monitoring and detection of animal well-being, suggesting factors for body and body weight problems, might enhance biological metrics in cattle. This might be performed by analyzing sounds, photographs, videos, and other data in real time. Data from remote sensors such as microphones, cameras, accelerometers, and thermometers can be used to gather reliable information when combined with animal IDs and other observations and put into algorithms [6]. Different sensors, such as radio frequency identification (RFID), accelerometers, load cells, and webcams, can be used to detect sudden changes in the activity, eating and drinking, physical condition, and health of animals [7]. Devices with the potential to measure physiological, immunological, and behavioral responses in livestock and various animal species are referred to as biosensors [8]. Biosensors are compact, portable, highly sensitive, fast, and can be extremely specific with a low chance of a false positive. They function in a variety of ways, including detecting changes in pH, ion concentrations, mass through particular hybridization, enzymatic reaction, functional loss, electrical potential change, color change, temperature, and so on. Novel biosensors have considerable advantages and uses in the management of livestock, including disease isolation and detection, reproductive cycle detection, health monitoring, and monitoring an animal’s physiological wellness through the examination of its surroundings [8].

The application of biosensors and wearable technology in animal health management is becoming increasingly important. These instruments can facilitate the early detection of diseases in animals, decreasing economic losses. A variety of sensors for animal health management are in various stages of commercialization across the world. Some approaches for properly detecting health status and illness diagnosis are solely relevant to humans, with only minor alterations or testing in animal models [9]. The current state of medical technology makes early illness detection difficult and laboratory testing for animals expensive [8]. There is a need for detection technologies that can anticipate when and in what group an incident is likely to occur, inform diagnosis and treatment options, and forecast potential repercussions on a specific community [8]. These tools can speed up the monitoring process and are not only accurate and sensitive for the parameters being analyzed, but they also can be dependable and simple to use. General farm monitoring may be made easier and more reliable by employing portable devices instead of conventional techniques such as taking notes, keeping a farm diary, or using simple equipment without data-sharing features. Many solutions have been developed for portable devices to reduce the labor associated with manually recording data [9].

Every year, new diseases that endanger the health of animals appear in the modern world. There are currently no viable, cost-effective diagnostic techniques for early disease identification in farmed livestock animals. Biosensing technologies have the ability to address these issues by providing novel diagnostic tools for the early detection of major health hazards in the agri-food animal sector [8]. Breeding is an essential component of animal husbandry. The ovulation cycle in cattle must be monitored in order to estimate the temporal frame for artificial insemination. Mastitis (both clinical and subclinical), metabolic disorders such as ketosis and acidosis, the animal’s reproductive status, and projected calving may all now be diagnosed using various technologies. As is known, automated milking systems (AMSs) pose various issues and possibilities for dairy producers. AMSs allow for the monitoring of milking frequency at the cow level, as well as quarter-level production and milk quality, which can contribute to the development of disease detection systems. Biomarkers such as plasma-hydroxybutyrate (BHB) and body condition score (BCS) might be beneficial not only in diagnosing illnesses, but also in evaluating cow pregnancy and re-production success [10]. BCS is a valuable approach for monitoring the relationships between nutritional management, reproduction, and metabolic diseases, and it facilitates farm management decisions [11]. BHB is one of the most useful indicators to diagnose ketosis [12]. It was found that BHB can be useful not only as an indicator for metabolic disorders, but also that the concentration of BHB in the blood has a negative phenotypic connection with the pregnancy rate at the first service [13]. Elevated BHB levels have been associated with uterine problems and delayed luteal activity [14]. Because it is simple, inexpensive, and has acceptable sensitivity and specificity, measuring inline lactate dehydrogenase (LDH) activity in milk in robots is a reliable indicator for the detection of subclinical mastitis [15]. As an inflammatory indication of mastitis, the milk enzyme LDH performed similarly to acute-phase proteins and somatic cell count, according to studies [16]. Using accurate diagnostic technology can enhance our understanding of the elements influencing the reproductive physiology of dairy cows [17]. In addition to assisting with reproductive control, the method enables the evaluation of luteal activity and its association with fertility through the analysis of frequent progesterone (mP4) data [10].

The focus of this review is to analyze emerging biosensing technologies that have the potential to impact livestock early disease diagnostics, management, and relevant procedures.

## 2. Importance of Early Diagnostic in Dairy Farming

Animal health management with biosensors is a new field that is gaining traction worldwide. Biosensors are increasingly being utilized in dairy farms to better monitor animal health and detect illnesses early on [9]. Making a correct diagnosis is a crucial intermediate step between identifying a disease’s root cause and treating it [18].

Continuous observation of behavioral and physiological indicators may make it possible to spot subtle alterations before they manifest as overt clinical symptoms. Cattle may benefit from early sickness detection by preventing disease development and improving treatment response [2]. Farm managers, veterinarians, and farmers themselves can use sensors to monitor animal movements, food intake, sleep cycles, and even shelter air quality. Big data-capable computers store and process raw data [19]. Proficient estrus identification is a constant issue for efficient reproductive performance in dairy herds, especially on farms that use artificial insemination (AI) [20,21]. In preparation for calving and milk production, dairy cattle experience many changes to their metabolism and bodies as they move from late gestation to early lactation. These profound alterations cause an increase in the likelihood of developing a wide variety of health problems, such as metritis, mastitis, ketosis, and displaced abomasum [22]. The absence of sickness is an important aspect of general animal health and well-being. Disease, lameness, and limb problems are already posing substantial challenges to the dairy industry. Lameness is uncomfortable, and animals in pain usually change their activity, stride, food, posture, and appearance from their regular behavior. The economic expenses associated with treatment, decreased milk output, decreased fertility, mortality, or removal from the herd may be lowered if a disease is diagnosed earlier [22]. The earlier adoption of intervention methods can be made possible by the earlier identification of cows at risk for health issues [19,22].

Devices that can be implanted in an animal’s body (thermography, pedometers) remain in the stomach (bolus) and provide owners with valuable information on the animal’s behavior and medical conditions. Using various devices prevents farm managers and veterinarians from spending unnecessary time on evaluating and inspecting animals. Veterinarians can catch diseases early on with wearable sensors (pedometers, GPS, milk analyzers, body condition scoring), which keeps animals from getting subclinically sick, clinically sick, or even dying. Owners can sort out sick animals in time to keep diseases from spreading through whole herds [9]. For instance, a decline in activity might be a sign of illness, and a decrease in time spent lying down could be a sign of discomfort or pain (pedometer, accelerometer) [23].

## 3. Innovative Tools in Farm Animals’ Early Disease Diagnosis

**Figure 1:**
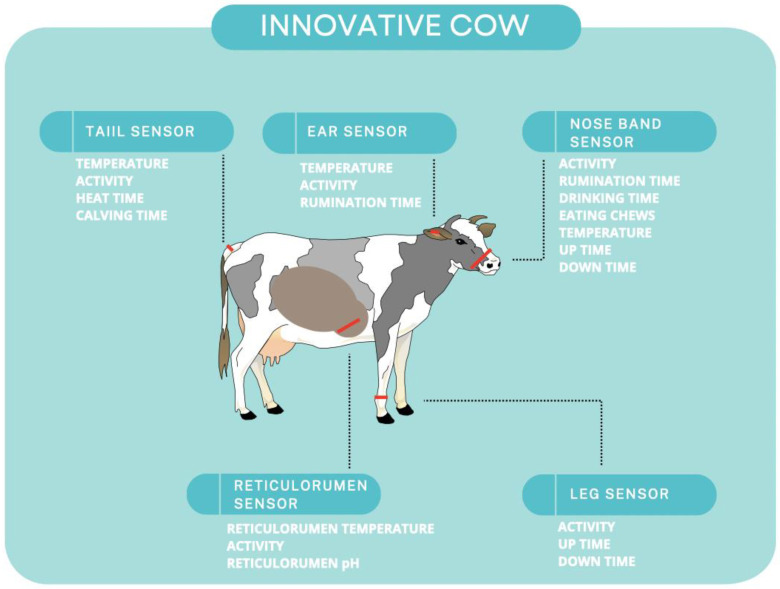
Innovative technologies applied to cows.

Farm management is being significantly influenced by technological breakthroughs, which are reducing physical labor, expenditures, and waste while increasing yields and profits. As a result of agricultural technological breakthroughs, a new farming method called “precision agriculture” has evolved [24]. Monitoring real-time autonomic responses (e.g., respiration rate, heart rate variability and heart rate, blood pressure, changes in peripheral blood flow) and defense-related reflexes with innovative biosensor equipment can aid in understanding how housing, nutrition, and genotype influence animals’ resilience to stressors. These sensors can contribute to the knowledge of factors that influence animal welfare and the creation of remedies (for example, husbandry techniques, genotype selection) that increase the welfare of livestock and companion animals. Wearable sensors can track feeding behavior, rumination behavior, rumen pH, rumen temperature, body temperature, laying behavior, animal activity, and animal location or placement [25]. Wearable or imprinted biosensors that allow remote data transfer could be significant in this rapidly evolving field [9]. There are many gadgets that can measure body temperature, behavior, and movement of the animal [9,26,27,28]. Sensors and wearable technology can be inserted into animals to detect the components of their body fluids such as sweat [9,29,30]. Cows are also fitted with commercially available biosensor collars to monitor the estrous cycle [9,31]. Figure 1 demonstrates examples of various technologies applied to cows.

### 3.1. Milk Analyzers

The use of technologies to evaluate physiological, behavioral, and production markers on individual animals in order to identify events of interest is included in the process of precision dairy monitoring [23]. Milk analytes can be used as biomarkers for diseases or reproductive status detection. For example, DeLaval Herd Navigator (DeLaval Inc., Tumba, Sweden) detects the amount of progesterone in milk. The program also recommends the ideal timing for insemination, names animals that need to be confirmed as pregnant, flags early abortions, and lists cows at risk for cysts and protracted anestrus. Other milking robots such as Lely Astronaut A4 (Lely Campus, Cornelis van der Lely an 1, 3147, PB, Maassluis, The Netherlands) can determine milk analytes and milk electrical conductivity. Blood biomarkers are useful indicators of animal health, although they have limited economic use. They might provide a great deal of information, especially because biomarkers can detect subclinical stages of illnesses even when the cow appears fully healthy and shows no visible indications of illness. An alternative to blood biomarkers can be milk biomarkers [10]. Sensor devices for determining the fat and protein content of milk are widely utilized on farms nowadays. According to each farm’s milking system, a unique sensor system is utilized. These sensors provide information on the health and fertility of cattle. Using milk analyzers, the estrous cycle and reproductive performance in dairy cows can be tracked [9]. There are various analyzers which may operate on different principles, some using chemical indicators and others based on spectroscopy [32].

#### 3.1.1. Somatic Cell Count

Mastitis-related milk production losses in dairy animals are economically significant [33]. Monitoring somatic cell count (SCC) concentrations in milk is the most widely used method for detecting mastitis, especially in its subclinical forms. When SCC values surpass the limit, the milk’s value plummets drastically. As a result, experts feel that SCC level is a significant parameter for evaluating udder health [34,35]. Despite the absence of clinical signs, subclinical mastitis is distinguished by an increase in SCC in milk. SCC is a useful udder health indicator since it counts the quantity of somatic cells (mainly desquamated epithelial cells, macrophages, and neutrophils) in milk. Aside from being used as a criterion for selecting dairy cows that are less susceptible to mastitis, the presence of SCC in bovine milk is a well-established indicator of mammary gland inflammation, which is strongly linked to the presence of a mammary infection. SCC has proven to be a useful indicator of decreased milk supply due to subclinical mastitis, which is present when the cell count is greater than 200,000 per milliliter [33,36,37,38,39].

Using cell staining methods and microscopy, the SCC concentration can be evaluated at the laboratory level. However, these approaches are time-consuming and call for specialized equipment and personnel [39]. Modern techniques for SCC in milk detection are much less laborious and time-consuming [39].

Automatic mastitis detection devices are examples of novel diagnostic processes that are field-adaptable, simple, and can rapidly provide results. There are many types of equipment such as the milk checker, Fossomatic meter (Hillerød, Denmark), Dramiski mastitis detector/Wykrywacz mastitis detector (Olsztyn, Poland), DeLaval cell counter (Tumba, Sweden), Afimilk mastitis detector (Kibbutz Afikim, Izrael), UdderCheck^®^ test (Moorestown, USA), and PortaSCC^®^ test (Moorestown, USA). They rely on either detecting physicochemical–biological alterations in milk or the udder or assessing biomarkers in body fluids (milk, serum) linked with mastitis [40].

It has been demonstrated that the diagnostic capacity of infrared thermography (IRT) is comparable to that of the California mastitis test, and it also distinguishes instances of clinical mastitis from those of subclinical mastitis. As a result, IRT has the potential to develop into a diagnostic tool that is both convenient and portable [40].

#### 3.1.2. Milk Progesterone

Fertility control needs close coordination between farmers and veterinarians, systematic examination of farm records, and reliable clinical data. Furthermore, low fertility might be considered a sign of poor health and well-being [41]. Breeding is an essential element of cattle husbandry. Detecting the ovulation phase in cattle is crucial for determining the best window for artificial insemination [8]. The presence of progesterone implies the existence of a functioning corpus luteum. As a result, it has been utilized for decades as a biomarker of reproductive efficiency. Milk progesterone is a potential non-invasive indicator for reproductive status in dairy cows since progesterone is transported from blood to milk [41]. Therefore, progesterone sensors could be useful sensor systems, despite the fact that little research has been carried out on the performance of such systems [42]. The Herd Navigation^®^ system (Tumba, Sweden), which integrates five sensing systems, including progesterone in milk, was developed for commercial usage in 2008. It detects progesterone levels in milk and recommends insemination times, animals for final pregnancy confirmation, early termination, and cows at risk for cysts and extended anestrus. In Denmark, estrus detection rates of 95–97% have been recorded, with much higher pregnancy rates (up to 42–50%) than traditional approaches [8]. Endocrine regulation is crucial for optimal fertilization throughout the follicular period. Because progesterone production is strongly related to embryonic development from the early stages of pregnancy, adequate monitoring of both pre-ovulatory decline and post-insemination elevation in milk progesterone can be used to detect animals with reduced fertility. Reduced milk progesterone concentrations, for example, around days 4–7 following insemination are related to low fertility and an increased chance of embryonic loss [41]. According to various studies, pregnant cows had higher quantities of milk progesterone in their milk samples for the first week following insemination [10].

Infrared spectroscopy is a fast, inexpensive, and user-friendly technology that can be used for research as well as online and offline milk analyses [24]. Near-infrared spectroscopy (NIRS) has long been used to measure the amount of milk components such as fat, protein, and lactose [43]. Numerous studies demonstrate that a near-infrared spectroscopy approach is a viable method for assessing individual milk progesterone levels. In fact, monitoring progesterone levels in milk is an efficient and cost-effective method for determining a cow’s reproductive status, detecting heat, and diagnosing pregnancy. Cows are milked twice or three times per day under standard dairy practices, implying that milk samples provide information on the current health of the herd/individual and may be collected and tested on a frequent basis without severely compromising the animal’s daily living [24]. In addition, it has been demonstrated that the physiological status of the animal affects the molecular structure of the water in milk; therefore, milk spectra can provide useful information regarding animal health and sickness [43].

### 3.2. Breath, Sweat and Saliva Analysis

Researchers have long been interested in disease detection by the identification of volatile organic compounds (VOCs), representing a non-invasive technique. Animals and humans both exhale and excrete VOCs in their secretions with breath, blood, feces, skin, urine, and vaginal discharges [44,45]. Gases such as hydrogen (H2) and methane (CH4), as well as volatile organic molecules such as fatty acids, which can serve as biomarkers for metabolic and pathologic processes, are examples of metabolites found in the breath. VOCs such as ketone bodies, ethanol, methanol, and exogenous compounds are commonly associated with blood glucose levels [46]. VOC analysis has been used to study bovine respiratory sickness, brucellosis, bovine tuberculosis, Johne’s disease, ketoacidosis, and normal rumen physiology in cattle [8,44].

The majority of sweat metabolite analysis biosensors were created with the intention of monitoring human health. These have been put to use to measure lactate levels and salt concentrations, and they have also been made portable (in belt form) to measure sweat [8]. Additionally, this sensor may be modified for use in measuring animal perspiration, particularly as an indicator of physical stress in animals [8,47].

Saliva collection for disease and other biochemical markers of physiological health is an appealing non-invasive alternative to blood sampling [48]. Because saliva contains both local and systemic components, it is a useful source of information regarding systemic processes occurring in the body, allowing for the evaluation of the organism’s physiological or pathological status. However, the use of saliva in the diagnosis of animal illnesses necessitates a detailed examination of its protein composition under various situations [49,50]. The technique is especially helpful for monitoring animals and diagnosing diseases because drawing blood from animals is thought to be a stressor and can affect the biochemical parameters being measured. Salivary biomarkers can be useful for several purposes, including disease early detection and diagnosis, decision support for managing animals, and disease progression monitoring [51]. There are other diagnostic applications for saliva, such as biomarkers from saliva being investigated for the detection of oral cancer [52]. According Mojsym et al., saliva can be a valuable diagnostic sample comprising possible indications of physiological and pathological conditions such as pregnancy status of cattle. Moreover, it can be useful for developing rapid tests from saliva [49]. In buffalo, there are attempts to determine estrous time using saliva biomarkers for more precise insemination planning [53]. Saliva analytes were explored for their relationships with lameness in another investigation. It was also anticipated that cows can change certain particles in their saliva, and some of these may reflect improvements in lameness after therapy [54]. Table 1 shows a summary of milk and other body fluid analysis and its benefits.

### 3.3. Wearable Devices for Animals

Portable electronic monitoring devices have the potential to transform intensive, large-scale dairy production by monitoring and managing cows on an individual basis. There has been a notable increase in the number of published research looking into the application of wearable electronic monitoring devices for use in commercial farming environments since the early 2000s [55].

#### 3.3.1. Head/Muzzle and Noseband Sensors

Three distinct kinds of biosensors can be employed to recognize the jaw movements that characterize cattle grazing behavior. These are electromyography sensors, mechanical/pressure sensors, and acoustic sensors [8]. Cattle grazing behavior necessitates close observation of each cow based on three crucial factors: the cow’s position, an analysis of its posture, and the cow’s motions, particularly its gait and jaw movement [8,56]. The amount of time an animal spends with its head in a downward posture is added to the sensor’s recorded data to calculate grazing time [57]. For instance, continuous observation of jaw motions can reveal information about diurnal grazing habits, animal health disorders, and forage deficiencies [58,59].

Animals adjust their behavior in response to stresses, social changes, and environmental changes, and these can cause illnesses. Because of the labor necessary for the continuous monitoring of big groups of animals, tracking this behavior on a large scale becomes impossible. When combined with proper output interpretations, wearable sensor technologies enable the simultaneous measurement of real-time physiological parameters in a herd on a large scale. As a result, wearable sensor technologies offer an advantage over traditional herd-based systems since data from wearable sensors can be evaluated instantly, allowing for a short reaction time [60]. For example, RumiWatch (RWS; ITIN + HOCH GmbH, Liestal, Switzerland), a noseband sensor that monitors feeding and rumination activity in dairy cows, was designed and tested as an effective scientific monitoring device for automated measurements of rumination behavior and activities. The correlations between direct observations and sensor readings reveal that the RumiWatch noseband sensor was effectively designed and validated as a scientific monitoring device for the automated detection of rumination and eating behaviors in stable-fed dairy cows [59,61]. Other examples of commercial sensors and their detected analytes are presented in Table 2. According to the findings of the study, the rumination and feeding activity monitoring system is an effective tool for predicting calving time under farm conditions. It was found that in primiparous and multiparous cows, lying bouts increased but rumination chews declined while predicting calving. Logistic regression and ROC analysis were used to assess the sensitivity (Se) and specificity (Sp) for predicting the commencement of calving within 3 h (Se = 88.9%, 85% and Sp = 93.3%, 74% for multiparous and primiparous cows, respectively) [62].

#### 3.3.2. Motion, Movement, and Behavior Sensors

Activity can be reduced in cattle afflicted with lameness or diseases such as bovine respiratory diseases (BRD). Energy conservation for immune system metabolic costs and indirect effects of fever and inflammatory responses to infection were proposed as biological underpinnings for reduced activity in ill animals [69]. There are plenty of technologies for motion, movement, and behavior analysis, such as accelerometer, pedometers, and global positioning system (GPS).

Accelerometers have been used in dairy farming systems for the detection of diseases such as mastitis and to detect estrus and locomotion problems [69]. Accelerometer reading changes can also be used to generate a benchmark level of activity, which can subsequently be recorded as calculated step counts or other movement indices such as activity ratios. Accelerometers have acquired popularity in beef cattle research because they allow for the continuous and long-term study of an animal’s mobility and behavior [69]. Moreover, ear-mounted sensors accurately identified grazing, standing, and walking in sheep with 94%, 95%, and 99% accuracy, respectively [70]. Commercially available accelerometer devices include the IceTag and IceQube products manufactured by IceRobotics, Ltd. (Edinburgh, Scotland, UK), designed and validated for use in cattle. Other commercial accelerometer products designed for use in cattle include CowScout (GEA Group, Dusseldorf, Germany), SCR (Allflex, Madison, WI, USA), Pedometer Plus (Madero Dairy Systems, Houston, TX, USA), GYUHO SaaS (Fujitsu, Fukuoka, Japan), and GP1 SENSR (Reference LLC, Elkader, IA, USA). Another accelerometer device that has been successfully used to quantify cattle behavior, the HOBO Pendant G (Onset Computer Corp., Bourne, MA, USA), requires the user to build a method of leg attachment, and data management is more complicated [69]. When compared to the accuracy reached with these devices (more than 90%), the accuracy of visual human observation is significantly lower [4].

Understanding how grazing animals migrate throughout pastures and what they do in each region is essential for developing management plans that will maximize the potential productivity of grazing systems and limit their negative impacts on the environment (nutrient losses to water and gaseous emissions). Using real-time global positioning system (GPS) tracking and biologging technologies, it is possible to perform remote monitoring of animals to look for any indications of illness or concerns regarding their well-being [71]. Animals on livestock farms can be monitored for their activity levels, which can provide useful information about the animals’ overall health and degree of care [72]. The global positioning system, radio tracking, and wireless local area network are now the most important technologies for monitoring livestock in the field; however, there are a few more tools (such as Bluetooth and ultrasound) that can be employed for indoor monitoring [71]. GPS collars equipped with activity sensors enable the distinction between foraging sites and those used for other activities such as sleeping or travelling [57,73,74]. Diverse studies conducted over the past decade have proved the utility of GPS telemetry devices for analyzing the behavior of cattle when combined with other devices/sensors. Combining GPS collars with activity sensors results in an effective method for tracking the whereabouts of grazing animals and determining animal behavior simultaneously. Real-time location systems have been created to pinpoint the position of an object within a particular area [6]. Although little research has been conducted on the behavior and movement of lame dairy cows on pasture, GPS technology may be useful in enhancing pasture-based systems’ automatic lameness diagnosis. According to Riaboff et al., severely lame cows spend 4.5 times less time grazing and nearly twice as much time resting in the laying position than their sound counterparts [75]. Additionally, GPS is used to forecast the behavior of ruminants and locate their location in pastures. This geolocation technique appears promising for identifying animals with a high frequency and a low mistake rate, despite the GPS’s inadequacy for forecasting behavior in a robust manner. Geolocated behaviors are especially intriguing for investigating changes in behavior connected to demanding events. The usage of data from accelerometers and GPS devices together is an approach that could prove to be both intriguing and beneficial in the process of researching how cows interact with their surroundings. These challenging scenarios can include heat stress, physical stress, resource depletion, restricted access to pasture, and other similar situations [76]. These characteristics could be used to improve the efficacy of existing lameness detection sensors in pasture-based systems [75]. Table 3 gives a brief summary of wearable sensors.

There are GPS systems that allow users to track and confine animals. Special attention has been paid to a solar-powered GPS collar-based virtual fence system (NoFence, Beatnfjordsra, Norway). The GPS position data collected by the collar are shown on an app that the farmer uses to create a grazing border map for cattle, sheep, or goats. When the animal gets close to the virtual boundary, the collar will begin to emit warning audio stimuli at a volume of forty decibels and a rising frequency of two to four thousand hertz. This will give the animal the time to change course to avoid such stimuli. When an animal passes the virtual barrier, it is considered to have “escaped”, and the auditory and the electric shock are turned off. Additionally, a push notification is delivered to the farmer’s mobile app when the animal has crossed the boundary [77]. GPS also can be promising in heat detection. Sheep estrus can be detected with global navigation satellite systems (GNSS) by monitoring a surge in activity levels followed by a return to “normal” patterns of behavior [70].

##### Pedometer

In animals, a pedometer (step counter) is a proven recording method for determining movement activity. Previously, it was mostly utilized for estrus detection [9]. Pedometers objectively measure an animal’s total number of steps and total distance traveled using an algorithm that calculates the steps. While pedometers are relatively simple to deploy and operate, the number of steps taken by each ruminant varies significantly depending on the day and ambient conditions. There could be a link between cattle distance traveled and stressful and unpleasant procedures; one study found that calves took fewer steps for four days after castration, while another found a link between calves’ stress and the number of steps they took after castration [6]. Other observations are made while recording active or lying behavior. The method is useful for the early identification of lameness in dairy herds. In one investigation, pedometers were used to detect lame calves before clinical indications appeared. Individual cows were examined and monitored, and it was shown that 92% of the cows acquired obvious lameness. When cattle were monitored using a pedometer, their hoof activity was reduced by at least 15% several days before the start of clinical lameness. The researchers concluded that pedometers are a beneficial tool for the early detection and treatment of the vast majority of cases of developing lameness [78].

### 3.4. Other Analyzers: BCS Camera, Infrared Thermography, Sensors of Bolus

#### 3.4.1. Infrared Thermography

Infrared thermography (IRT) is method for diagnosing and assessing pain since it indicates physiological changes [6,79]. Thermography can be used to identify and determine thermal abnormalities in animals by identifying a rise or decrease in the surface temperature of the skin [80,81]. IRT is a technique that is frequently utilized in the field of veterinary medicine to diagnose conditions such as infection, lameness in horses, and mastitis in both sheep and cattle, and to determine scrotal temperature in buffalo [70]. IR thermography is a noninvasive method that monitors emitted infrared radiation and displays the data as a thermogram, which is a visual representation of an object’s surface temperature. Each pixel in the thermogram represents the recorded surface temperature of an object. The data can be displayed in grayscale or color. The warmest areas are displayed in white or red on a color scale, while the coldest areas are shown in black or blue. When an animal is stressed, the hypothalamic–pituitary–adrenocortical axis is engaged, and heat is created as a result of increased catecholamine and cortisol concentrations, as well as blood blow reactions, resulting in changes in heat generation and heat loss from the animal. As a result, this technique may be beneficial as a general stress indicator [80]. Changes in surface temperature patterns, particularly those generated by changes in blood flow, can be utilized to detect inflammation or damage associated with illnesses such as foot lesions. Thermal (color) variations represent thermal gradients, which represent changes in skin temperature induced by underlying illnesses. Thermography has the advantage of measuring heat emissions without having direct physical contact with the surface. Because of its high sensitivity, it is useful when paired with other exact data (such as pedometer, accelerometer activity). In general, thermography works best in concert with other modalities rather than as a replacement for them. Thermography frequently detects physiological changes before they emerge as clinical symptoms, allowing for early diagnosis and treatment [79,80]. According research, there was no statistically significant difference in foot temperature amongst different illnesses, but there was a difference between sick and healthy cows [81].

To reduce economic losses caused by lameness, preventive interventions and early diagnosis of lesions are required [78]. The use of infrared thermography to detect lameness in cattle has increased in recent years, owing to its non-invasive qualities, ease of automation, and continued cost savings [79,80]. Lameness in dairy cows has negative effects not only on dairy cow welfare and milk production, but also on reproductive ability and death rates [78]. Early diagnosis of a foot lesion can help reduce the negative impact of lameness and boost treatment success, and is likely to be useful in preventing future pathological development [80].

Thermography can also be employed in research of various diseases such as musculoskeletal affections and ocular temperature assessment in calves for early illness diagnosis. This instrument has also been used to identify mammary gland inflammation [79]. Infrared thermography has been shown to be useful in assessing udder health and detecting quarters with subclinical mastitis [34]. Mastitis raises the warmth of the udder before clinical symptoms appear. Furthermore, after inoculating lactating cows with *Escherichia coli* in various locations of the udder, Pezeshki et al. noticed a 2–3 °C rise in udder surface temperature [60]. Berry et al. demonstrated that thermography of the udder can be a useful diagnostic tool for detecting mastitis in dairy cattle. Because the temperature rises considerably three days before ovulation, using thermography to identify cow estrus can increase pregnancy chances in regular or quiet estrus [82]. An Australian study involving Holstein cattle found that a fall in vulva and muzzle temperature 48 h before ovulation correlates to corpus luteum regression, and an increase in temperature 24 h before ovulation corresponds to the period of estrus [82]. Thermography could also be used to detect estrus in sheep and goats [77]. Zaninelli et al. investigated the potential of IRT in the diagnosis of mastitis in their 2018 study and discovered that it corresponds well with the somatic cell count [83]. Thermography has been demonstrated to distinguish between clinical and subclinical mastitis in both large and small ruminants, with diagnostic sensitivity and specificity comparable to the California mastitis test (CMT). As a result, with further adjustments and advancements, farmer-friendly and non-invasive infrared thermography has the potential to become a useful and practical instrument for use on farms in the future. Individual determinations for each mammary compartment can be made, with an increase in local temperature suggesting the presence of inflammation, if it exists [34].

In cases of stress, fertility, welfare, metabolism, health, and illness detection, the animal’s surface temperature can be used as an indicator trait to accurately measure an animal’s physiological state [6,83]. Thermal infrared sensors have shown that there is a strong link between changes in the temperature around the eyes and changes in the temperature of the body’s core. Temperature readings from different parts of an animal’s body give information about its health and allow for quick decisions about its welfare (e.g., isolating animals with higher body temperature or managing the internal temperatures of animal housing units) [6]. Salles et al.’s study shows that among the body regions tested, IRT frontal temperature had the strongest correlation with rectal temperature, while the temperature–humidity index is substantially related with forehead and right and left flank temperature [84]. Another investigation found out that setting the illness threshold for filthy feet at 27 °C correctly detected 80% of feet with lesions and 73% of feet without lesions [85]. Although this method is sensitive in identifying changes in the temperature patterns of animals, it may not be adequate to pinpoint their causes [83].

As the livestock industry’s reliance on automated systems grows, technologies that can be implemented into these systems to monitor animal health and welfare must be developed. Infrared thermography (IRT) is one such technology that has been utilized for monitoring animal health and welfare and has the potential to be incorporated into automated agricultural systems through automation [86].

#### 3.4.2. Bolus Sensors

Bolus sensors are primarily intended to detect variations in ruminal temperature, which can indicate a change in animal physiological states [87]. Ruminal temperature decreases in response to drinking and eating events, and it rises in response to increased body temperature. Monitoring changes in ruminal temperature and activity can help spot anomalous behavior, the estrous cycle, and infections early [87,88,89].

Metabolic illnesses (rumen acidosis, hypocalcemia) and other diseases that cause fever and pain have an effect on the amplitude and frequency of ruminal contractions in cattle [6]. Wireless intraruminal bolus sensors have been devised to monitor the temperature and pH levels of the rumen and reticulum via the esophagus. The availability of boluses for measuring reticuloruminal pH for purchase has simplified the evaluation process greatly. These boluses wirelessly transfer pH information to a central processing location on a regular basis, simplifying the evaluation procedure. An example of bolus sensors can be seen in Table 2.

The pH level of the ruminal fluid is one of the most straightforward indicators that ruminal acidosis is present. The ruminal pH can be measured in real time by wireless pH probes that are put in boluses within the rumen. In addition to the pH of the rumen, various markers for either ruminal acidosis or subacute ruminal acidosis can be determined in blood, urine, feces, or milk. These can be used to diagnose either condition [41].

Other studies, while using bolus sensors, have shown that cows with higher rumen pH (6.22–6.42) can emit 46.18% more methane than cows with lower ruminal pH [90]. Cantor et al.’s study revealed that reticulorumen temperature is an accurate predictor of well-being factors in cows such as daily herd water intake and inflammation [91].

#### 3.4.3. Body Condition Score

Body condition score (BCS) is an evaluation of the cow’s fat stores and indicates the cow’s overall energy balance. The amount of fat mobilization in cows with a higher BCS is higher [22]. There is a substantial correlation between body condition score (BCS) and many reproductive and lactational performances in small ruminants, making BCS an important indication of their well-being [77]. BCS at calving and its fluctuations during lactation have been found to have an impact on the health and fertility of high-yielding dairy cows. When a cow’s condition deviates from acceptable BCS standards, the occurrence of metabolic disorders, infertility, and lameness increases [10].

Having a high or low BCS can have unfavorable effects on milk production, illness, and reproduction [92]. However, because of its subjectivity and slowness, manual BCS evaluation is rarely used outside of experimental settings or large farms with many ruminants [77]. The BCS scale typically consists of a five-point scale with increments of either 0.25 or 0.5 points [93]. Reduced early lactation dry matter intake and milk production, as well as an increased risk of metabolic problems, are all connected with a calving BCS of 3.5 or below on a five-point scale. On a scale from 0 to 5, a BCS of 3.0 to 3.25 is considered ideal for calving. When it comes to production and reproduction, lower calving BCS is connected with lower rates, while greater calving BCS is associated with an increased risk of metabolic diseases [94]. Consequently, measuring and regulating the body condition and obtaining optimal BCS at various phases of lactation are crucial for maintaining or enhancing the performance and welfare of cattle, especially dairy cows [95].

Anatomical points from the rear end of the dairy cow (such as the hooks and tail-head area), which are located in an area of the cow where changes in subcutaneous body reserves are visually more evident, are visually evaluated in order to assign a score according to a standardized scale in the process of visual body condition scoring [95]. In the past decade, numerous proposals for agricultural three-dimensional (3D) vision systems that are based on optical two-dimensional (2-D) and 3D sensors were developed [96]. Recent years have seen an increase in the use of 3D sensors in the applications of BCS. These sensors bring more information about the body’s surface than 2D-based or thermal image-based systems [93]. Three-dimensional cameras have recently advanced in technology and could provide novel feed management solutions for dairy farms. Portable ASUS Xtion Pro sensors (ASUSTeK Computer Inc.) can be used to capture 3D photos of cows’ rear ends [97]. There is also a commercially available automatic BCS camera for use on dairy cows’ heads (DeLaval Body Condition Scoring, BCS DeLaval International AB, Tumba, Sweden) [92,98]. This camera system uses a radio frequency identification reader to individually identify cows that have been fitted with transponders. It also enables multiple BCS measurements to be taken within a single day. The camera software records individual daily BCS values for each scoring session and reports a daily trimmed, 7-day rolling average of BCS [95]. The BCS camera system produces accurate BCS ratings between 3.00 and 3.75; however, the magnitude of low and high BCS scores is typically miscalculated.

Time of flight (ToF) cameras are quickly becoming one of the most common types of sensors used to acquire 3D data. ToF systems use either visible or near-infrared (NIR) light, and the reflected light is received by smart pixel sensors, which then measure the amount of time it takes for the light to return. It was demonstrated that the characteristics extracted from the dorsal and posterior sections achieved 100% accuracy of the projected BCS within a 0.5 point deviation of the actual BCS) [93].

Studies have shown that BCS can not only be a metabolic disease marker, but can also be associated with production, health, and reproduction. Antanaitis et al. found that BCS is associated with pregnancy success because the BCS (+0.29 score) and mP4 (10.93 ng/mL) of the pregnant cows were higher compared to the group of non-pregnant cows [99].

#### 3.4.4. Animal Surveillance through Video and Imaging

Livestock illness prediction and abnormal behavior management both benefit greatly from automated tracking systems. Animals can be monitored in a number of ways, some of which are manual, some of which include wearable devices, and some of which involve computerized tracking systems. However, relying on human observers to keep tabs on animals is labor-intensive and not always reliable. The level of intelligent livestock management can be greatly improved with the help of computer vision technology, which provides a non-contact and low-cost technique to track cattle. Researchers have put much time and effort into determining how to achieve the level of autonomous monitoring of animals shown in the video.

Face recognition is another emerging technology that is sweeping the globe. One study compares three loss functions used in human face recognition paired with RetinaFace-mobilenet, and the results suggest that the proposed CattleFaceNet beats others with an identification accuracy of 91.3% and a processing time of 24 frames per second (FPS) [100]. Li et al. suggested a method for recognizing individual dairy cows based on an image of the animal’s tail and head. This approach retrieved form characteristics from the tail head image’s region of interest using Zernike moments and categorized them using four classifiers [101]. Gaber et al. proposed a muzzle-based livestock recognition strategy that employed the Weber local descriptor and the Adaboost classifier for each head photo to identify the head of cattle [102]. In other studies to evaluate cow lameness, color edge detection and bilateral projection were utilized to detect the region of the cow’s knees. For cow identification and recognition, these approaches rely on color and contour characteristics. However, the strong reliance on hand-crafted characteristics precludes these systems from being applied to complicated settings. Deep learning approaches and Convolutional Neural Network models (e.g., Fast R-CNN, Faster R-CNN, YOLO, SSD, Mask R-CNN, AlexNet, VGGNet, and ResNet) have recently achieved substantial advancements in image identification and recognition through end-to-end feature learning [103,104,105,106,107,108,109,110,111,112]. With the release of a new large-scale cow dataset with about 50,000 annotated cow face detection data points and approximately 18,000 cow recognition data points, cow face detection and recognition have been improved. There is also a framework for cow face recognition that combines the detection and recognition models to improve recognition performance. The experimental results show that the proposed technique is superior. The detection accuracy is 98.3%, while the accuracy of cow facial recognition is up to 94.1% [113]. A sheep study showed results suggesting that the method proposed in that study outperforms the others, with a recognition precision of 89.12%, and it was discovered that incorporating the biometrics of the sheep face can significantly boost the network’s recognition capacity [114]. In Kumar et al.’s study, experimental findings on a muzzle point picture database were described. Their solution achieved 93.87% identification accuracy, demonstrating its superiority over other existing machine-learning-based recognition systems [115].

Su et al. conducted a study with the Dairy Goat Dataset (DG-dataset) containing 200 dairy goat motion videos with a total of 161,000 frames of images randomly collected from the farm. The study shows that their algorithm was successful in locating and tracking a single dairy goat [116]. Video tracking of animals helps to recognize ill animals or strange behavior in animals without human contact.

#### 3.4.5. Electronic Nose for Estrus Detection

The MENT-EGAS prototype used electronic nose (EN) technology (ten unspecified chemical metal-oxide sensors) to detect estrus by direct sampling of odor from the perineal headspace. In cycling cows, principal component analysis (PCA) revealed effective discrimination between proestrus and estrus, as well as estrus and metestrus. Based on these findings, it was demonstrated for the first time that direct sampling of the perineal headspace using an EN device during milking may correctly detect estrus in dairy cattle [20]. In this study, the MENT-EGAS prototype (Patent No. WO2010099800A2) provided by AIRSENSE ANALYTICS GmbH (Schwerin, Germany) was used to detect emanated odor changes from the perineal headspace of the cows. Another study described an effective electronic nose system created using polyaniline-based sensors doped with various acids for determining estrus in female cattle. Disposable swabs for material gathered from the perigenital area and vagina of the animals were employed in the suggested olfactory technology, eliminating rectal manipulation, as well as disposable sensors, providing simplicity of handling and health safety for the estrus determination procedure. The results demonstrated that the olfactory system reliably detected estrus, optimal AI moment (12 h after estrus detection), and diestrus (corpus luteum phase) in cows, and that this information could be utilized to efficiently suggest the ideal AI time in calves [117]. Thermography and its measured analytes and other electronic devices are reviewed on Table 4.

## 4. Innovations for Common Procedures

At present, innovations are becoming inseparable from animal husbandry. Mastitis (both clinical and subclinical), metabolic diseases such as ketosis and acidosis, the reproductive state of the animal, and predicted calving now can be determined by various technologies. Two features of milk quality—the somatic cell count (SCC) and the appearance of obviously abnormal milk in cases of clinical mastitis—are used to detect mastitis in milk [119]. Some technologies and their functions in disease and behavior diagnostics are shown in Table 5. The real-time detection of beta-hydroxybutyrate from blood or milk is used to determine the animals’ energy balance and can be greatly aided by nano-biosensors. One of the metabolic illnesses, subclinical ketosis, raises the chance of developing clinical ketosis, lowers milk production, impairs reproductive ability, and has a negative energy balance during the transition phase, all of which have an adverse economic impact [120].

## 5. Conclusions and Future Directions

The primary goal of precision livestock farming is to generate reliable data using biosensors and run it through intelligent software systems to create value for the farmer, the environment, and the animals in the form of improved animal health and welfare, increased productivity and yields, and lower costs while minimizing environmental impact. Sensor-based technology has made important contributions to lowering animal stress, improving animal well-being, and thereby eliminating economic losses. By forecasting future disease outbreaks, the early identification of physiological reactions can assist farmers in taking targeted interventions to reduce pressure on their animals, increase animal welfare, and avert performance losses. Future technological advancements will lead to the identification of biomarkers for specific health and welfare concerns at a far earlier stage. Precision livestock farming aims to create a management system based on automatic, continuous, real-time monitoring and control of all aspects of livestock management, including reproduction, animal health and welfare, and the environmental impact of livestock production. Monitoring animal behavior without upsetting the animal is another component of management. Farmers can employ wearable sensors to detect illness early, reducing animal deaths. Farmers and veterinarians can also destroy sick animals before they spread disease to the entire herd of cattle, if they plan ahead. Through satellites and smartphones, the incorporation of revolutionary diagnostic and disease detection systems utilizing biosensors would keep cattle and the agricultural business one step ahead of unseen diseases. When biosensors are used to detect diseases early, the epidemiological curve can be moved to the left because quick action can be taken to stop the spread of the disease and its negative effects on production, society, and the economy. An early warning system for more effective livestock health management can be created by shortening the amount of time it takes to receive findings when diagnosing infectious disease biomarkers on a farm in a manner that is in real time.

## Figures and Tables

**Table 1:** Body fluid analysis and its benefits.

Technology	Benefits of Use	Reference
Milk progesterone	Milk progesterone is a potential non-invasive indicator of reproductive status in dairy cows	[41]
Somatic cell count	SCC has proven to be a useful, non-invasive indicator of subclinical mastitis	[36]
Breath, Sweat and Saliva analysis	Biomarkers for metabolic and pathologic processes are examples of metabolites found in the breath. VOCs such as ketone bodies, ethanol, methanol, and exogenous compounds are commonly associated with blood glucose levels. Saliva collection is a non-invasive alternative to blood sampling	[9,44]

**Table 2:** Information about some of the commercial sensors.

Sensor	Method	Detected Analytes	Reference
RumiWatch (Itin + Hoch GmbH, Liestal, Switzerland)	The RW system comes with software for controlling the sensor (RW Manager) and studying unprocessed data (RW Converter). The RW sensors, which include a noseband pressure sensor, a three-axis accelerometer to track three-dimensional head motions, and a data logger, are built into a halter that fits the head of each particular animal. The noseband pressure sensor, which is mounted in a belt on the animal’s nose bridge, is connected to a tube filled with propylene glycol to detect jaw movements. As the animal moves its jaw, pressure within the tube varies, and this information is recorded with a 10 Hz resolution. Approximately 100 days of raw data logging were covered by the battery life.	Different pressure signatures of jaw motions, which are then detected and categorized into prehension bites, mastication chews, and rumination chews	[57]
Ear tag–based accelerometer system (Smartbow GmbH, Weibern, Austria)	The ear tag has an acceleration sensor, a radio chip, and a temperature sensor for calibration and it can monitor rumination and detect estrus and localization.	Rumination, estrus, and current localization	[63]
MoonSyst (Moonsyst International Ltd.: P.O. Box 1329, Kinsale, Co., Cork, Republic of Ireland)	System captures rumen data in real time. The bolus is meant to be readily ingested and will remain in the rumen (particularly the reticulum) throughout the animal’s life. System sends data from the animal to specialized cloud-based servers via a communication gateway. Farmers may use the Mooncloud software application to view information from anywhere, anytime. The bolus can be used on animals weighing more than 350 kg. Once implanted, the bolus interacts with a gateway over a large geographical region.	Heats, monitor health conditions, activity, rumen temperature and movement	[64]
SmaXtec (SmaXtec animal care GmbH, Graz, Austria)	The rumen bolus accurately monitors direct, informative values inside cows’ reticulum. The boluses are given once and require no further maintenance. The data from the boluses are read out by the readout devices with an integrated Internet connection and promptly transferred to the cloud. The pH and temperature variation data are gathered with an analogue-to-digital converter (A/D converter) and stored in an external memory chip. This indwelling system may be simply orally supplied to an adult cow due to its dimensions (length: 12 cm, width: 3.5 cm, weight: 210 g), and its particular construction makes it shock-proof and resistant to rumen fluid.	pH, ruminal temperature, cow activity, drinking, eating, rumen behavior	[65]
Body Condition Score Camera (DeLaval, International AB, Tumba, Sweden)	Body condition score system is based on a 3D camera that records certain areas of the animal: from above, the rear part of the back from the short ribs to the tail end. When a cow moves in front of the camera, the system recognizes the movement and records photographs of the cow; it then selects the best image of the cow from the video clip. The 3D camera employs light coding technology to project a pattern of infrared ray dots on the cow’s back. Following that, the distances between these specific dots are measured; the company claims that a 3D picture of the back is created, and an algorithm translates the image information into a body condition score.	BCS	[10]
CattleEye (Cattle Eye Ltd., Belfast, UK)	Camera is above the exit gate of a milking parlor. It records video of each cow as it exits the milking parlor. If a sort of gate or RFID system provides ID information, use it. Artificial intelligence systems in the cloud analyze video to uniquely identify the cows and track their wellness, among other things. System allows tracking the health and performance of cows in real time. It includes a dashboard that monitors and visualizes a variety of vital indicators at the herd and cow levels.	Cow identification	[66]
Cainthus (© 2022 Ever.Ag, Frisco, TX, USA)	Smart camera system that monitors animal behavior and farm activities 24 h a day, seven days a week, 365 days a year. It is artificial intelligence that converts visual input from cameras into real-time insights. These insights are provided daily on any farm device, phone, tablet, or computer. The information provided is accurate and unbiased. This technique is easily scalable, does not require any hardware on the cows, and requires extremely minimal maintenance in comparison to other solutions.	Animal behavior	[67]
BROLIS Herdline (Vilnius, Lithuania)	The analyzer examines the composition of each cow’s milk during each milking. This “mini-spectroscope” is installed in the milking stalls or milking robot in the milk line and does not use additional reagents and does not require special maintenance. The analysis of protein, fat, lactose, and electrical conductivity provides a proper evaluation of the health, productivity, and economic efficiency of dairy cattle. The data collected during milking are processed in real time and can be viewed using the BROLIS HerdLine application.	Milk fat, protein, lactose, milk electrical conductivity	[32]
HeatWatch (HeatWatch^®^ DDx, Inc., Denver, CO, USA)	A tiny radionic transmitter is linked to a pressure sensor in a stiff plastic box implanted in a nylon packaging that is glued to the cow’s tail hair in the sacral region. The device is activated by the weight of the mounting animal for a minimum of 2 seconds, after which the transmitter sends the breeding approval signal to the system along with the animal’s identification. In general, this device’s assessed performance ranges from 37% to 94%.	Heat detection	[68]

**Table 3:** Wearable sensors and their benefits.

Technology	Benefits of Use	Reference
Head/muzzle and noseband sensors	Noseband sensor was designed and validated as a scientific monitoring device for the automated detection of rumination and eating behaviors. It can be executed without contact with the animal.	[59]
Motion, movement, and behavior sensors	Accelerometers, pedometers, and GPS tracking all can be used to monitor animal behavior. Active time can predict heat; prolonged laying time can signal diseases such as mastitis, ketosis, and lameness. GPS helps to locate animals on the farm.	[69,72,76]

**Table 4:** Other sensors and their benefits.

Technology	Benefits of Use	Reference
Infrared Thermography	Determine thermal abnormalities in animals by identifying a rise or fall in the surface temperature of skin. Infrared thermography is a noninvasive method that monitors infrared radiation emitted from the body. Inflammation, stress, calving, and heat can be evaluated. Thermography can detect physiological changes before they emerge as clinical symptoms.	[79,80,81]
Bolus Sensors	Wireless intraruminal boluses without constant contact, can measure and analyze ruminal and eating behavior, examine ruminal pH.	[6,41,118]
Body Condition Score Cameras	Tracking BCS can help reduce postpartum disease percent; it helps to notice obese or poor health animals. When it comes to production and reproduction, lower calving BCS is connected with lower rates, while greater calving BCS is associated with an increased risk of metabolic diseases	[92,93,94,96]
Cattle Face Recognition	Face analysis can help to identify pain, unwell animals, locate, identify, and select animals on the farm.	[102,113]
Electronic Nose for Estrus Detection	Can detect estrus by direct sampling of odor from the perineal headspace.	[117]

**Table 5:** Technologies for animal status determination.

Disease/Status of Cow	Technology for Diagnosis	Analytes	Reference
**Mastitis**	Image processing, spectroscopy, electrical conductivity, biosensors, SCC sensors, tri-axial accelerometers, pedometers, spectroscopy	Temperature, lying behavior, eating behavior, milk analytes (fat, protein, electrical conductivity), rumination time, somatic cell count (SCC), milk pH, milk yield	[25,32,34,121,122,123,124,125,126,127]
**Metritis/Endometritis**	Tri-axial accelerometer, electronic feeding system	Eating, drinking time, rumination, activity, laying time,	[2,25,128,129,130]
**Ketosis**	3D cameras, spectroscopy, milking robots, accelerometers	body condition score, BHB, milk analytes (fat, protein), milk yield, activity, rumination behavior	[10,13,131,132]
**Acidosis**	Three-axis accelerometers, angular velocity sensors, pH meter, milking robots	Milk yield, milk analytes (fat, protein) activity, rumination behavior, walking behavior, feeding behavior	[118]
**Lameness**	Tri-axial accelerometers, pedometers, video observations, accelerometers, rumination sensor	Walking behavior, feeding behavior, rumination, activity, and laying time have been linked to lameness	[25,80,133,134,135]
**Heat**	Tri-axial accelerometers, pedometers, video observations, accelerometers, spectroscopy, chemical analysis, electronic nose, acoustic sensors	Activity, milk analytes (progesterone), odor from the perineal headspace, pression, friction, rumen movement	[20,21,25,117,136,137]
**Pregnancy**	Milking robots, radioimmunoassay, enzyme immunoassay, accelerometers, pedometers	Milk progesterone, activity, temperature	[136,137,138,139,140]
**Calving, dystocia**	Intravaginal thermometer, tri-axial accelerometers, pedometers, video observations, accelerometers, rumination sensor, infrared thermometry (IRT imaging)	Body temperature, activity, rumination time, laying time	[137,141,142]

## Data Availability

Not applicable.
